# Gelatinous macrozooplankton diversity and distribution dataset for the North Sea and Skagerrak/Kattegat during January-February 2021

**DOI:** 10.1016/j.dib.2022.108493

**Published:** 2022-07-30

**Authors:** Louise G. Køhler, Bastian Huwer, José Martín Pujolar, Malin Werner, Karolina Wikström, Anders Wernbo, Maria Ovegård, Cornelia Jaspers

**Affiliations:** a Centre for Gelatinous Zooplankton Ecology and Evolution, National Institute of Aquatic Resources, Technical University of Denmark, Kemitorvet 202, 2800 Kgs. Lyngby, Denmark; b National Institute of Aquatic Resources, Technical University of Denmark, Kemitorvet 201, 2800 Kgs. Lyngby, Denmark; c Institute of Marine Research, Department of Aquatic Resources (SLU Aqua), Swedish University of Agricultural Sciences, Turistgatan 5, S- 453 30 Lysekil, Sweden

**Keywords:** IBTS, Jellyfish, Comb jelly, gelatinous zooplankton, NIS, Non-indigenous species

## Abstract

This data article includes a qualitative and quantitative description of the gelatinous macrozooplankton community of the North Sea during January-February 2021. Sampling was conducted during the 1^st^ quarter International Bottom Trawl Survey (IBTS) on board the Danish R/V DANA (DTU Aqua Denmark) and the Swedish R/V Svea (SLU Sweden), as part of the ichthyoplankton investigation during night-time. A total of 147 stations were investigated in the western, central and eastern North Sea as well as the Skagerrak and Kattegat. Sampling was conducted with a 13 m long Midwater Ring Net (MIK net, Ø 2 m, mesh size 1.6 mm, cod end with smaller mesh size of 500 µm), equipped with a flow meter. The MIK net was deployed in double oblique hauls from the surface to c. 5 m above the sea floor [1,2]. Samples were visually analysed unpreserved on a light table and/or with a stereomicroscope or magnifying lamp within 2 hours after catch. A total of 13,510 individuals were counted/sized. Twelve gelatinous macrozooplankton species or genera were encountered, namely the hydrozoan *Aequorea vitrina, Aglantha digitale, Clytia* spp., *Leuckartiara octona, Tima bairdii, Muggiaea atlantica*; the scyphozoans *Cyanea capillata and Cyanea lamarckii* and the ctenophores *Beroe* spp., *Bolinopsis infundibulum, Mnemiopsis leidyi, Pleurobrachia pileus*. Abundance data are presented on a volume specific (m^−3^) and area specific (m^−2^) basis. Size data have been used to estimate wet weights based on published length-weight regressions (see Table 1). For the groups i) hydrozoan jellyfish, ii) scyphozoan jellyfish, iii) ctenophores, as well as iv) grouped gelatinous macrozooplankton, spatial weight specific distribution patterns are presented. This unpublished dataset contributes baseline information about the gelatinous macrozooplankton diversity and its specific distribution patterns in the extended North Sea area during winter (January-February) 2021. These data can be an important contribution to address global change impacts on marine systems, especially considering gelatinous macrozooplankton abundance changes in relation to anthropogenic stressors.

## Specifications Table

**Table:** 

Subject	Biodiversity Marine Biology Zoology
Specific subject area	Data describe the gelatinous macrozooplankton community in the North Sea and Skagerrak/Kattegat during 1^st^ quarter 2021. Included are abundance and size data for jellyfish (Hydrozoa, Scyphozoa) and Ctenophora.
Type of data	3 Tables 8 Figures 1 Appendix (raw data table)
How the data were acquired	Ship based, night-time sampling of gelatinous macrozooplankton with a 13 m long Midwater Ring Net (MIK net, 2 m diameter, mesh size 1.6 mm, mesh size cod end: 500 µm). The entire unpreserved samples were analysed for gelatinous macrozooplankton. Species-specific abundance and size data have been recorded and were used to estimate wet weights from literature sourced length-weight regressions (Table 1). For the Swedish dataset, *Aglantha digitale* abundances were estimated from abundance groups (see methods section). Sub-sampling was conducted for very abundant taxa and sub-sampling precision was assessed in the laboratory from re-counts of very abundant species at ten stations.
Data format	Raw and analysed data
Description of data collection	Samples from MIK double oblique net casts were visually analysed immediately after catch without preservation. Species were identified and measured with either an electronic or conventional caliper on a light table, under a magnifying lamp with dark background or using a stereomicroscope. Counts per species and sampling station were standardized to volume (individuals m^-3^) and area (individuals m^-2^) specific abundance estimates using calibrated flow meter values and reported maximum net depths during the plankton hauls.
Data source location	National Institute of Aquatic Resources, Technical University of Denmark, DTU Aqua 2800 Kgs. Lyngby Denmark Institute of Marine Research, Department of Aquatic Resources (SLU Aqua), Swedish University of Agricultural Sciences, 453 30 Lysekil Sweden
Data accessibility	Deposited on zenodo with doi: 10.5281/zenodo.6821876, are provided as appendix 1 and available via this data repository link: https://doi.org/10.5281/zenodo.6821876.

## Value of the Data

• This dataset is important for assessing the biodiversity and distribution of native and non-indigenous gelatinous macrozooplankton in the North Sea and Skagerrak/Kattegat during winter (Q1 2021). This dataset can help to address the impact of rising winter temperatures on the diversity, distribution and abundance pattern of gelatinous zooplankton.
• The data can be used as supplement to existing data compilations in order to establish a baseline for future monitoring of gelatinous macrozooplankton in the North Sea and Skagerrak/Kattegat, including the so far largely neglected winter period.
• The here described methodology and protocol has been applied by Denmark and Sweden during the 1st quarter IBTS night-time plankton investigation. The dataset highlights the importance of close international collaboration between zooplankton, ichthyoplankton and fisheries scientists to gain insights into diversity and distribution patterns of native and non-indigenous gelatinous macrozooplankton species, which have so far largely been neglected.
• Quantification of gelatinous macrozooplankton during targeted fisheries and ichthyoplankton surveys with standardized protocols represent a unique platform and thereby resource in order to target jellyfish-fish interactions and explicitly address the long standing questions if gelatinous zooplankton biomass changes in relation to anthropogenic stressors.

## 1 Data Description

**Fig. 1:**
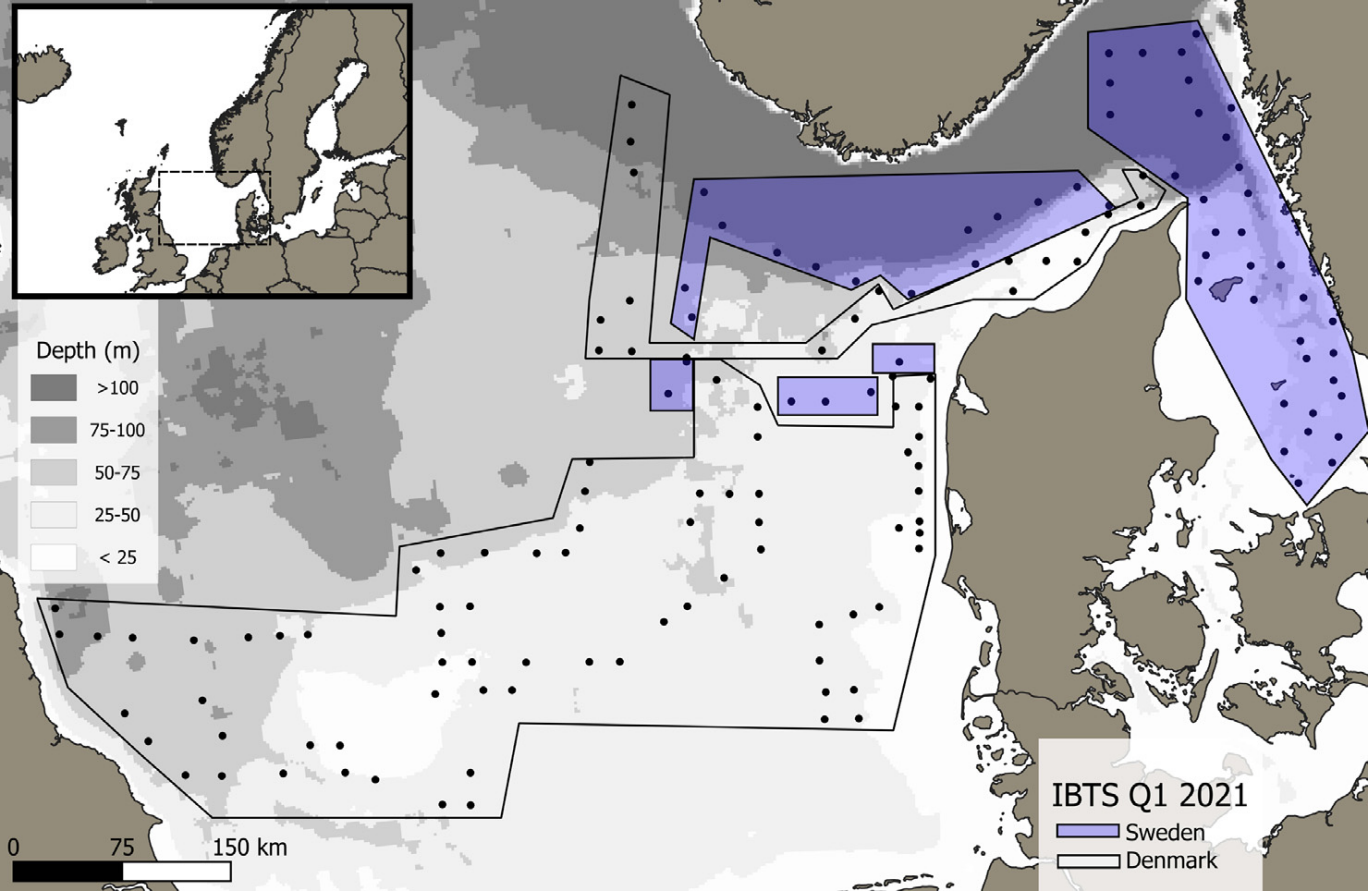
Investigation area of the North Sea, including Skagerrak as well as Kattegat, where gelatinous macrozooplankton was collected during the 1^st^ quarter IBTS surveys for Swedish (blue) and Danish (grey) investigations, January-February 2021. Sampling stations indicated by black dots.

**Fig. 2:**
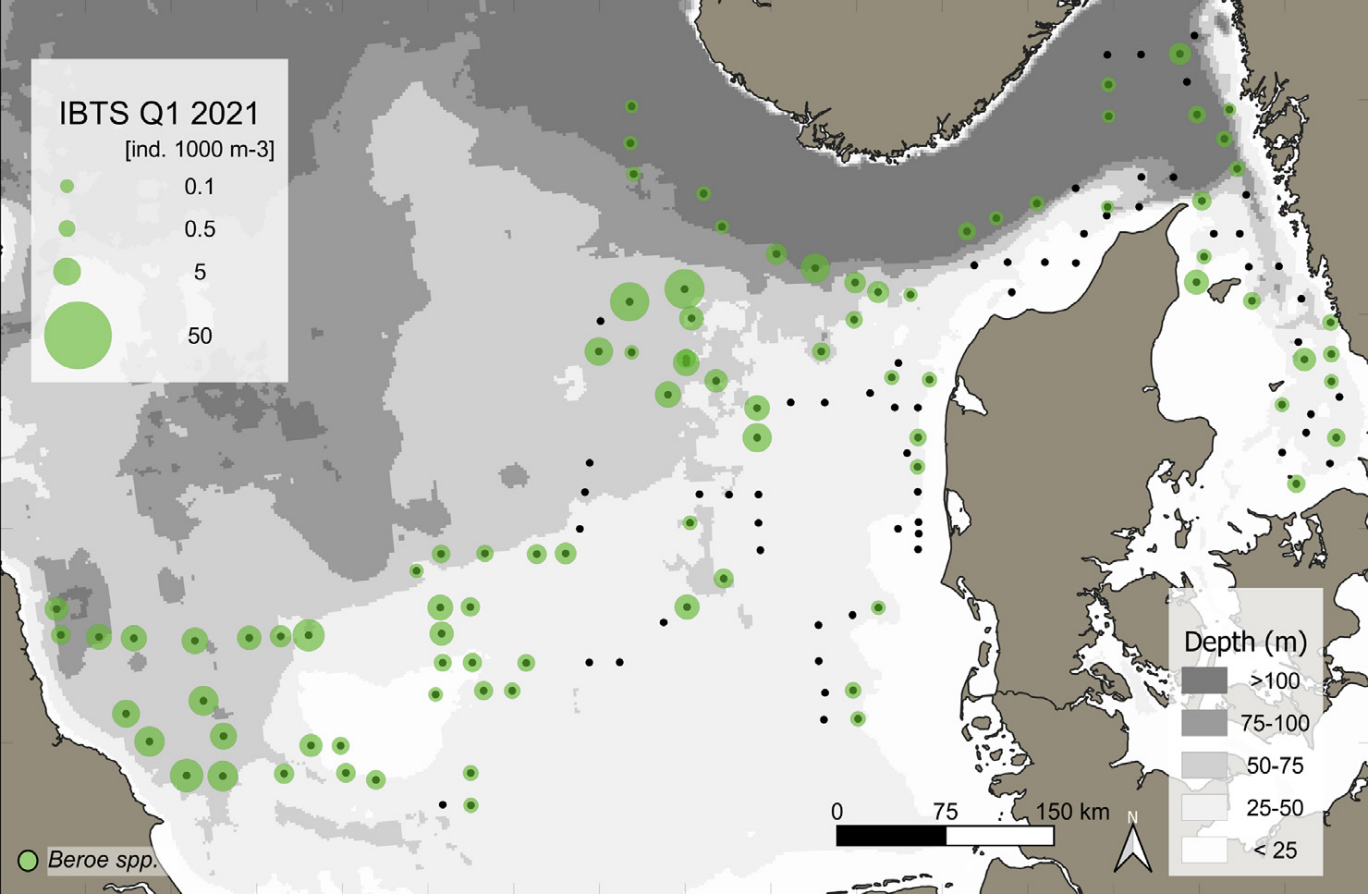
Distribution and abundance (individuals 1000 m^−3^) patterns of the ctenophore *Beroe* spp. in the North Sea and Skagerrak/Kattegat during January - February 2021. Black dots indicate sampling stations.

**Fig. 3:**
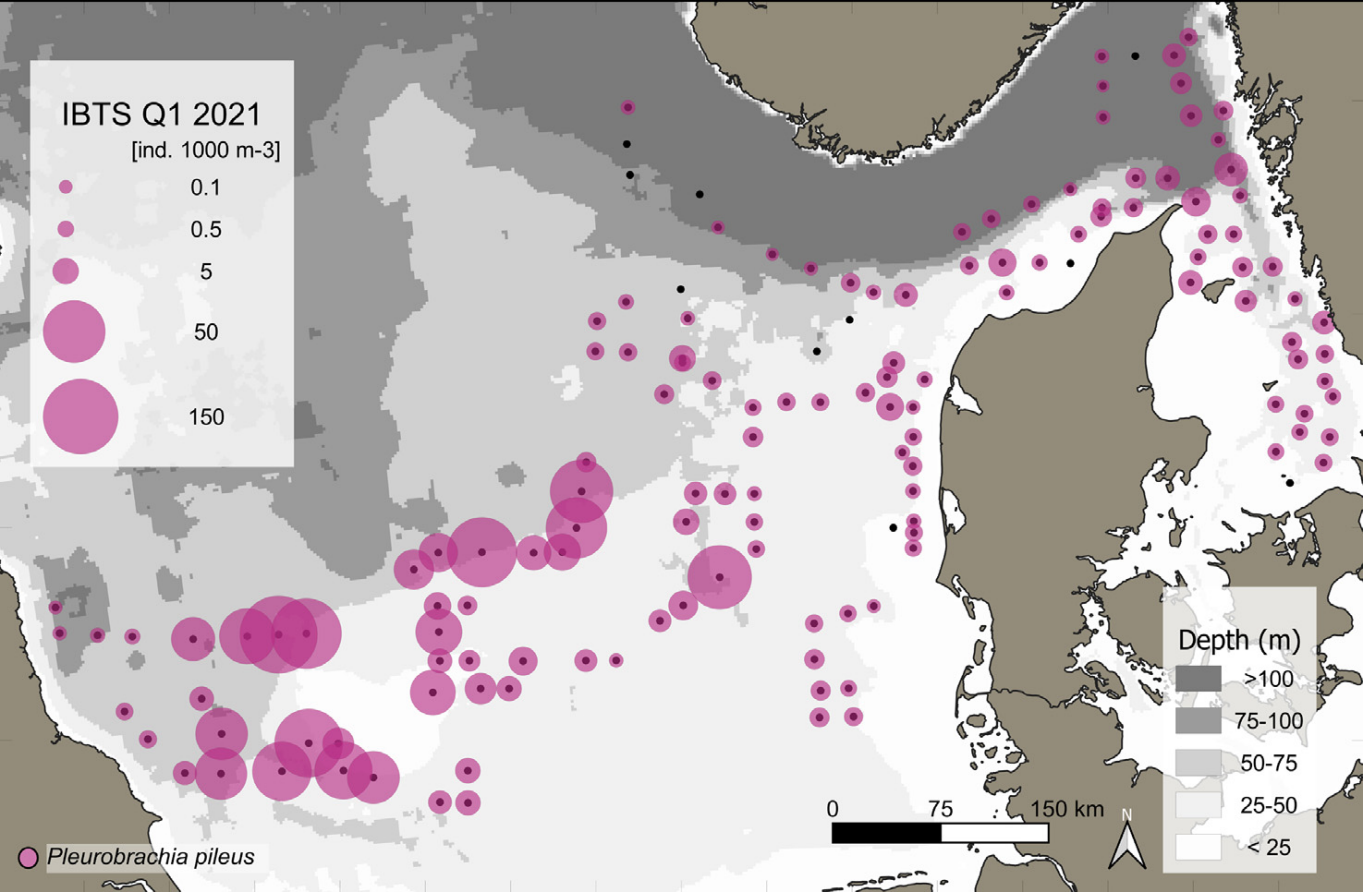
Distribution and abundance (individuals 1000 m^−3^) patterns of the ctenophore *Pleurobrachia pileus* in the North Sea and Skagerrak/Kattegat during January - February 2021. Black dots indicate sampling stations.

**Fig. 4:**
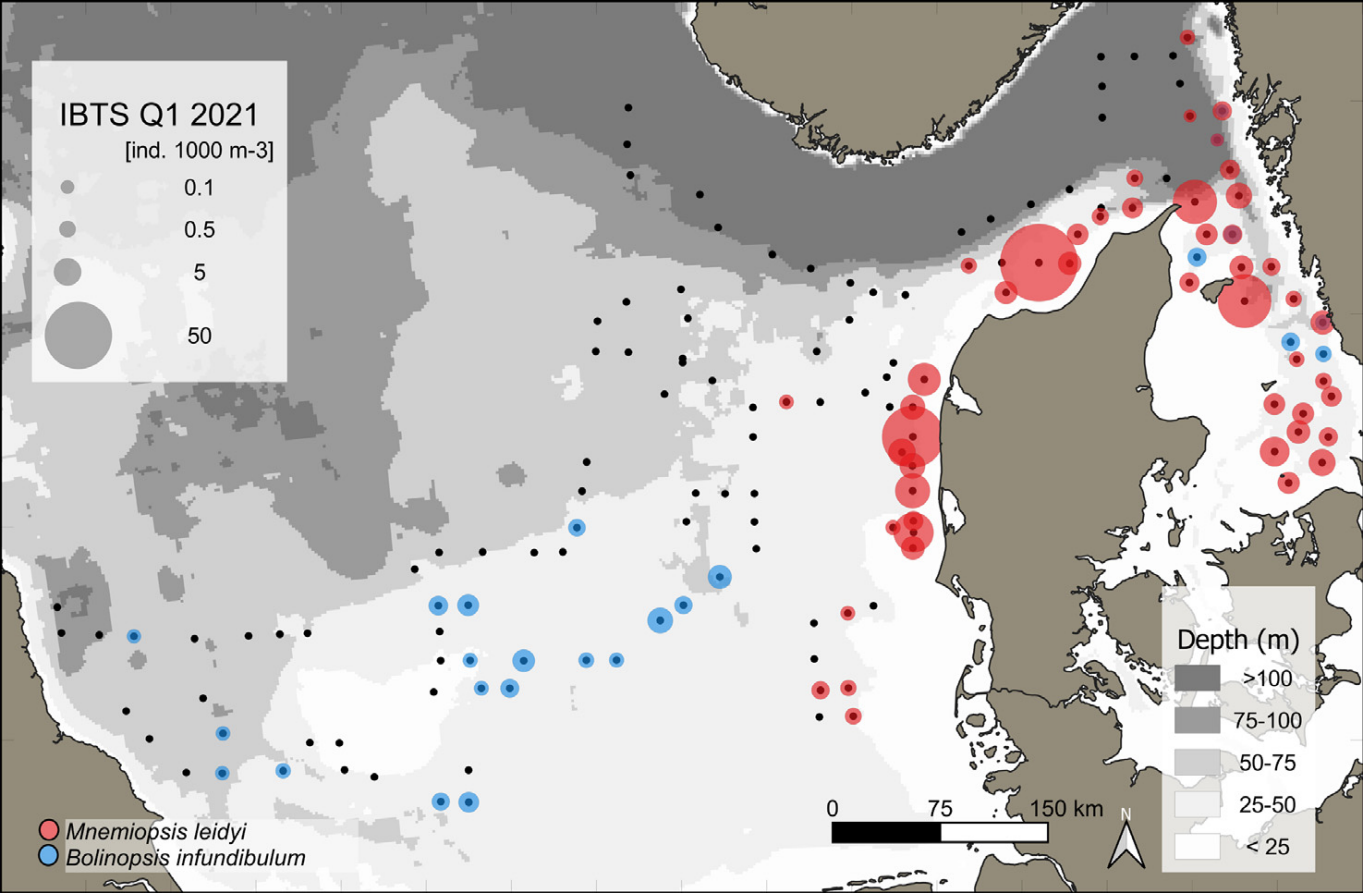
Distribution and abundance (individuals 1000 m^-^^3^) patterns of the non-indigenous ctenophore *Mnemiopsis leidyi* (red) and the native ctenophore *Bolinopsis infundibulum* (blue) in the North Sea and Skagerrak/Kattegat during January - February 2021. Black dots indicate sampling stations.

**Fig. 5:**
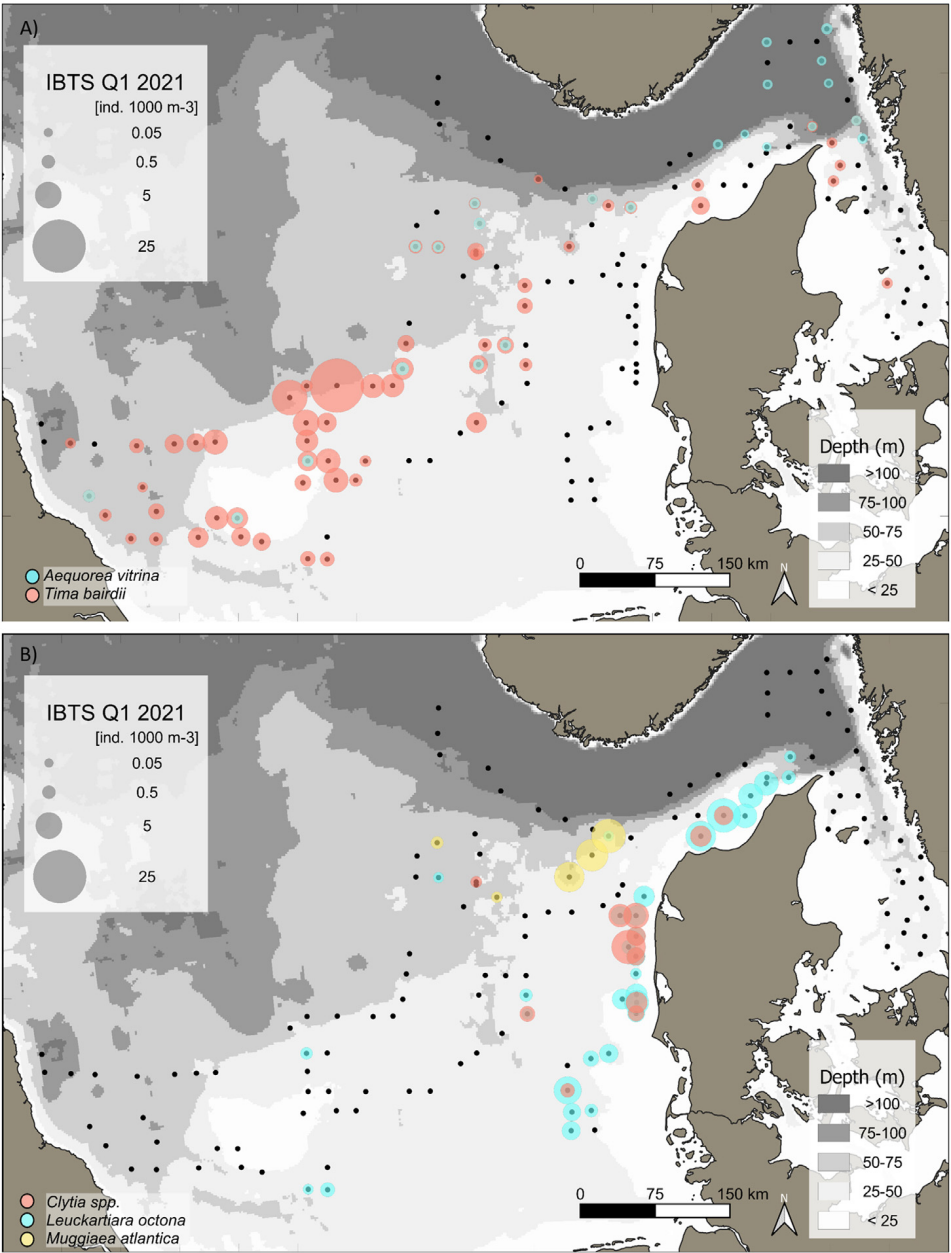
Distribution and abundance (individuals 1000 m^−3^) patterns of the hydrozoan species *Aequorea vitrina* (blue) and *Tima bairdii* (red) (A) and *Clytia* spp. (orange), *Leuckartiara octona* (turquois), *Muggiaea atlantica* (yellow) (B) in the North Sea and Skagerrak/Kattegat during January - February 2021. Black dots indicate sampling stations. Sweden did not quantify this small hydromedusae, which were not abundant during their surevy.

**Fig. 6:**
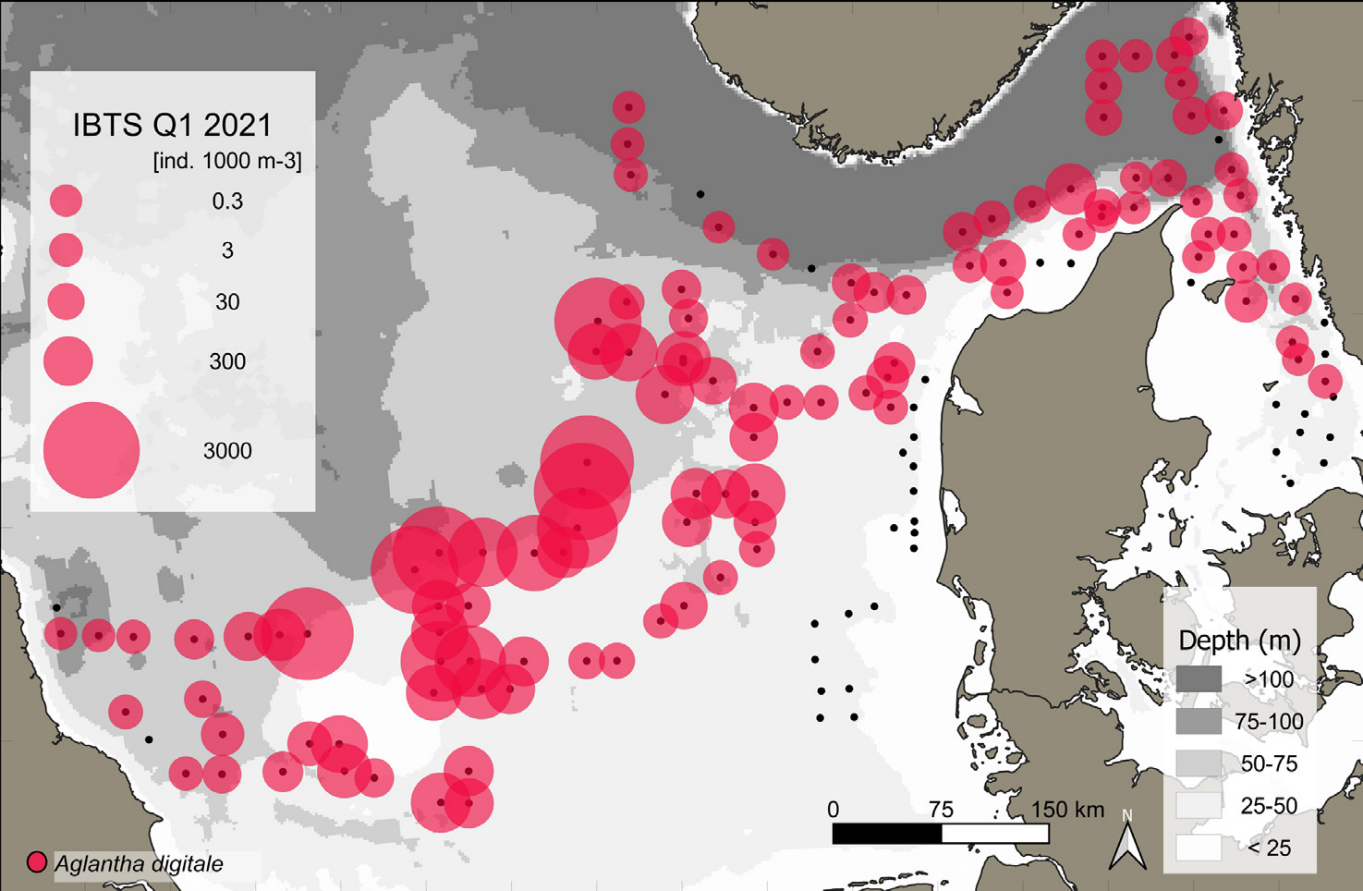
Distribution and abundance (individuals 1000 m^−3^) patterns of the hydrozoan jellyfish species *Aglantha digitale* (red) in the North Sea and Skagerrak/Kattegat during January - February 2021. Black dots indicate sampling stations.

**Fig. 7:**
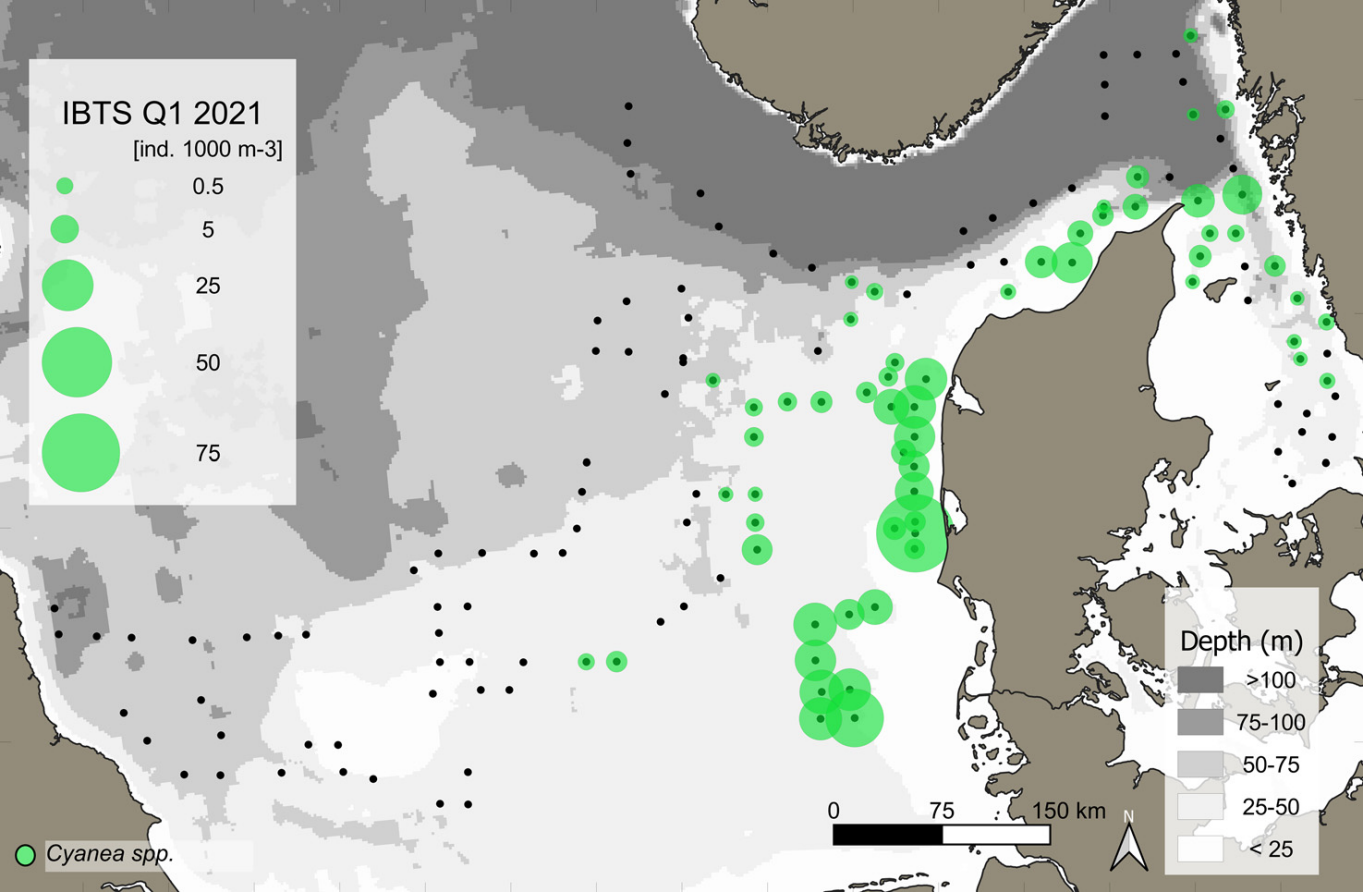
Distribution and abundance (individuals 1000 m^−3^) patterns of the scyphozoan jellyfish species *Cyanea* spp. (green) in the North Sea and Skagerrak/Kattegat during January - February 2021. Black dots indicate sampling stations.

**Fig. 8:**
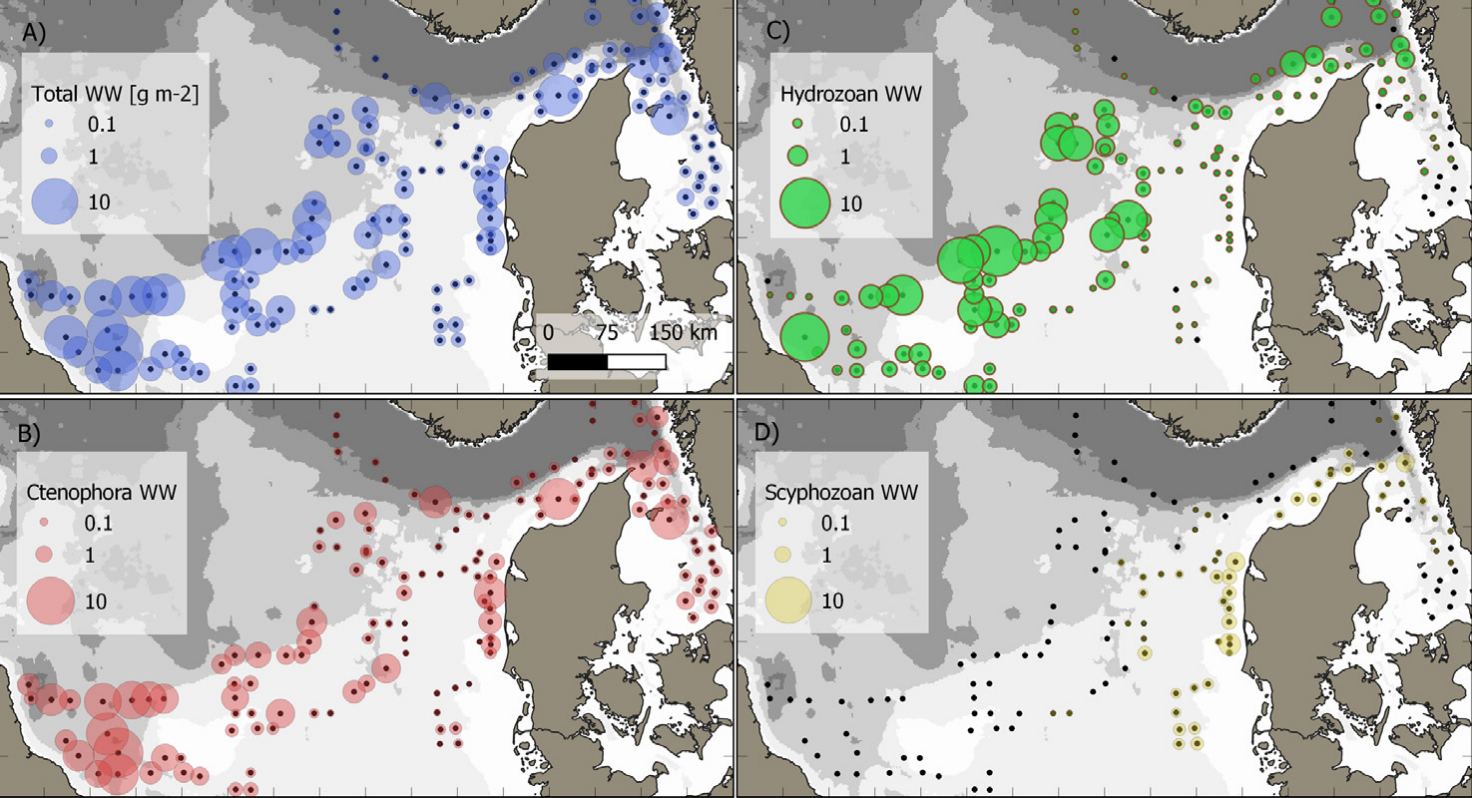
Biomass distribution of the gelatinous macrozooplankton community encountered in the North Sea and Skagerrak/Kattegat during January - February 2021. Black dots indicate sampling stations. Biomass is expressed as wet weight (g, m^−2^) for A) the total gelatinous macrozooplankton, B) grouped ctenophore, C), hydrozoan jellyfish, and D) scyphozoan jellyfish community encountered during the IBTS Q1 surveys of Denmark and Sweden.

This data article presents a description of the gelatinous macrozooplankton community of the western, central, and eastern part of the North Sea including the Skagerrak as well as the Kattegat, which belongs to the extended Baltic Sea region. Data were collected at 147 stations during January - February 2021 as part of the Danish and Swedish contribution to the International Bottom Trawl Survey (IBTS) (Fig. 1). The dataset consists of species-specific spatial distribution, abundance and size data as well as estimated wet weights (see Table 1 for length-weight regressions) for gelatinous macrozooplankton in the North Sea area during winter 2021 (Appendix 1 with raw data, data shared in repository zenodo doi: 10.5281/zenodo.6088227, Fig. 2, Fig. 3, Fig. 4, Fig. 5, Fig. 6, Fig. 7 for distribution maps). Additionally, wet weight distribution pattern of grouped hydromedusae, scyphomedusae, ctenophora as well as grouped gelatinous macrozooplankton are provided (Fig. 8). Species or genera specific characteristics for the Swedish (Table 2) and Danish (Table 3) cruises are summarized, including total counts, average and maximum abundance on a volume (individuals 1000 m^−3^) and area (individuals m^−2^) specific basis as well as average, minimum, and maximum sizes. A total of 13,510 specimen were analysed and sized, including four ctenophores: *Beroe* spp., *Bolinopsis infundibulum, Mnemiopsis leidyi, Pleurobrachia pileus*, six hydrozoans: *Aequorea vitrina, Aglantha digitale, Clytia* spp*., Leuckartiara octona, Tima bairdii, Muggiaea atlantica* and two scyphozoans: *Cyanea lamarckii and Cyanea capillata*. Due to the presence of many early life stages (ephyra) of *Cyanea* and the difficulty to separate them to species level, both species have been grouped into *Cyanea* spp. For the Swedish dataset, hydrozoans apart from *A. digitale* (abundances estimated in abundance groups 1-4), *T. bairdii* and *A. vitrina* were not encountered in large densities and have not been quantified. *A. digitale* was the most abundant species encountered during both surveys, which is why their abundances have been estimated using different approaches. In the Swedish dataset, *A. digitale* abundances have been estimated from abundance groups, while for the Danish dataset, abundances have been estimated from sub-samples. To assess sub-sampling effects 10 samples with the highest *A. digitale* abundances from the Danish survey were re-analysed in the laboratory to confirm abundance extrapolations. We found that differences in abundance estimates from sub-sampled *A. digitale* during the cruise (n = 652 individuals analysed) and in the laboratory (n = 1153 individuals analysed) were negligible and differed by only 1.8 ± 3.2 %. The calicophoran siphonophore *Muggiaea atlantica* was collected at 5 stations in the western North Sea during the Danish survey, consisting of polygastric stages only. Size and weight data were used to assess wet weights (g) for all groups using published length-weight regressions (Table 1).

**Table 1:** Length-weight regressions from literature to estimate wet weight (WW, g) from diameter (D), height (H) or length (L) for all gelatinous macrozooplankton species / genera encountered during the IBTS cruises in the extended North Sea area during Q1 2021.

Phylum/Class	Order	Species	Regression	Reference
Hydrozoa	Leptothecata	Aequorea vitrina*	DW (mg) = 0.03 x D (mm)^2.3^	Møller and Riisgård (2007) [11]
	Trachymedusae	*Aglantha digitale*	WW (mg) = 0.33 x H (mm)^1.95^	Runge et al. (1987) [12]
	Leptothecata	*Clytia* spp.*	DW (mg) = 0.093 x D (mm)^1.46^	Lucas et al. (1995) [13]
		Tima bairdii*	DW (mg) = 0.03 x D (mm)^2.3^	Møller and Riisgård (2007) [11]
	Anthoathecata	*Leuckartiara octona*⁎⁎	W (µgC) = 0.443 x H (mm)^3.10^	Daan (1986) for *Sarsia tubulosa*[5]
	Siphonophorae	*Muggiaea atlantica*	Av. WW (g) = 0.0195 g ind^−1^	Rutherford and Thuesen (2005) [7]

Scyphozoa	Semaeostomeae	*Cyanea* spp*.*	WW (g) = 0.185 x D (cm)^2.77^	Båmstedt et al. (1994) [14]

Ctenophora	Beroida	*Beroe* spp*.*	WW (mg) = 1.77 x L (mm)^2.23^	Finenko et al. (2001) [15]
	Lobata	*Mnemiopsis leidyi*⁎⁎⁎	V (mL) = 0.0009 x L_oa_ (mm)^2.84^	Jaspers et al. (2015) [8]
		*Bolinopsis infundibulum*	same as *M. leidyi*	Jaspers et al. (2015) [8]
	Cydippida	*Pleurobrachia pileus*	WW (mg) = 0.682 x L (mm)^2.52^	Mutlu and Bingel (1999) [16]

⁎ Dry weight (DW) estimated to represent 4% of the wet weight WW [4];

⁎⁎ Carbon weight = 0.5% of WW [6];

⁎⁎⁎ Displacement volume (mL) and wet weight (g) = 1.0g cm^−3^ [see 9,10]

**Table 2:** Gelatinous macrozooplankton characteristics from the Swedish IBTS survey Q1 2021 in the North Sea and Skagerrak/Kattegat with total counts per species (n), volume specific (individuals 1000 m^-3^) and area specific (individuals m^-2^), average (av.) and maximum (max.) abundance data as well as size characteristics, including minimum sizes (min). Smaller hydromedusae were not counted apart from *Aglantha digitale*, for which abundances were estimated from abundance groups at 41 stations. For *Aglantha digitale* and *Cyanea* spp. average size was used for weight estimates, see methods section for details (n.d. = no specific data).

			Abundance (1000 m^-^^3^)		Abundance (m^-^^2^)		Size (mm)		
Class	Species	n	av. ± SD	max.	av. ± SD	max.	av. ± SD	min.	max.
Ctenophora	*Beroe* spp.	510	1.7±3.16	16.5	0.1±0.19	0.8	24.5±10.9	5	70
	*Bolinopsis infundibulum*	19	0.35±0.29	0.9	0.02±0.01	0.03	14.8±6	5	20
	*Mnemiopsis leidyi*	478	3.3±5.04	19.6	0.11±0.19	0.84	21.8±6	10	40
	*Pleurobrachia pileus*	1177	2.92±2.9	14.5	0.16±0.19	0.79	13±3.7	5	30

Hydrozoa	*Aequorea vitrina*	20	0.15±0.09	0.4	0.01±0.003	0.02	154±31.5	100	220
	*Tima bairdii*	18	0.22±0.2	0.8	0.01±0.01	0.03	49.2±4.9	40	60
	*Aglantha digitale*	41 (19690)	50.5±110.2	628.3	3.2±6.8	34.9	n.d.	n.d.	n.d.

Scyphozoa	*Cyanea* spp.	225	1.7±3.2	13.3	0.08±0.17	0.8	n.d.	n.d.	n.d.

**Table 3:** Gelatinous macrozooplankton characteristics from the Danish IBTS survey Q1 2021 in the North Sea with total counts per species (n) including extrapolated counts from sub-samplings (in brackets) as well as volume specific (individuals 1000 m^-^^3^), area specific (individuals m^-^^2^), average (av. ± SD) and maximum (max.) abundance data. Size characteristics include minimum size (min.). Sub-sampling was conducted for *Aglantha digitale* (49 stations), *Pleurobrachia pileus* (18 stations), *Mnemiopsis leidyi* and *Cyanea* spp. (1 station each). In addition to the species mentioned in the table, twenty Siphonophore stems of the family Physophoridae (*Physophora hydrostatica*) were caught at 19 stations (see raw data).

			Abundance (1000 m^−3^)		Abundance (m^−2^)		Size (mm)		
Class	Species	n	av. ± SD	max.	av. ± SD	max.	av. ± SD	min.	max.
Ctenophora	*Beroe spp.*	738	2.4±2.7	13.7	0.14±0.17	0.72	29±12.9	4.6	220
	*Bolinopsis infundibulum*	86	1.1±1.1	4	0.05±0.05	0.17	21.3±2.2	19	63
	*Mnemiopsis leidyi*	609 (654)	8.3±16.5	68.2	0.19±0.33	1.3	20.1±4.8	5.6	46
	*Pleurobrachia pileus*	3054 (9133)	21.5±36.6	161	1.09±2.0	10.3	14±2.8	3	27

Hydrozoa	*Aequorea vitrina*	10	0.25±0.1	0.47	0.01±0.01	0.02	239±58.5	110	300
	*Aglantha digitale*	4178 (17888)	507±774	3059	26.7±47.5	205	7.45±1.46	2	17
	*Clytia spp.*	116	2.6±2.5	9.4	0.07±0.06	0.24	7.5±4.6	2	45
	*Leuckartiara octona*	278	2.1±2.2	9.3	0.06±0.06	0.24	6.6±1.25	2.3	16
	*Muggiaea atlantica*	139	5±4.4	9.4	0.26±0.25	0.57	6.6±0.8	4	15
	*Tima bairdii*	518	2.3±3.9	25.9	0.1±0.2	1.4	46.6±10.3	5	84

Scyphozoa	*Cyanea spp.*	1317 (1371)	8.6±11.7	62.8	0.25±0.3	1.44	19.9±8.4	3	70

Description of all encountered gelatinous macrozooplankton species, visualization of their distribution patterns and outlining species-specific characteristics of the Swedish (Skagerrak/Kattegat) and Danish (North Sea) data are provided in detail below.

***Beroe* spp.** (Ctenophora) were found throughout the North Sea, Skagerrak and Kattegat, but most abundant in the western and northern part of the North Sea (Fig. 2). *Beroe* spp. were caught at 90 stations. Mean (± SD) *Beroe* spp. abundances were 0.1 ± 0.17 and 0.14 ± 0.17 individuals m^−2^ with a maximum of 0.77 and 0.72 individuals m^−2^ for the Swedish and Danish surveys, respectively (Table 2 and 3). This ctenophore had an overall mean (± SD) and maximum abundance in the entire dataset of 0.13 ± 0.17 and 0.8 individuals m^−2^ or 2.15 ± 2.72 and 14.38 individuals 1000 m^−3^ (see raw data). Separated by surveys, sizes ranged between 5 to 70 mm with a mean size of 24.5 ± 10.9 mm for the Swedish and 5 to 220 mm with a mean size of 29 ± 12.9 mm for the Danish survey, respectively (Table 2 and 3).

***Pleurobrachia pileus*** (Ctenophora) was the second most abundant species in both surveys. This ctenophore was present at 137 stations or >93% of all stations (Fig. 3). *P. pileus* was ubiquitous with an overall mean (± SD) and maximum abundance of 0.74 ± 1.65 and 10.3 individuals m^−2^ or 14.6 ± 30.4 and 161 individuals 1000 m^−3^ (see raw data). Mean (± SD) *P. pileus* abundances were 0.16 ± 0.19 and 1.1 ± 2 individuals m^−2^ with a maximum of 0.79 and 10.3 individuals m^−2^ for the Swedish and Danish surveys, respectively (Table 2 and 3). *P. pileus* abundance 1000 m^−3^ was 2.92 ± 2.9 (max: 14.5) and 21.5 ± 36.6 (max: 161) for the Swedish and Danish surveys, respectively (see Table 2 and 3). Average sizes were 13 ± 3.7 mm (range: 5 – 30 mm) for the Swedish and 14 ± 2.8 mm (range: 3 – 27) for the Danish datasets (Tables 2 and 3).

***Mnemiopsis leidyi*** (Ctenophora) were found in the eastern part of the North Sea along the coast of Denmark and in the Skagerrak and Kattegat, while being absent from the central and western North Sea (Fig. 4). *M. leidyi* were found at 48 stations. This ctenophore had an overall mean (± SD) and maximum abundance in the entire dataset of 0.15 ± 0.27 and 1.3 individuals m^−2^ or 5.6 ± 12 and 68.2 individuals 1000 m^−3^ (see raw data). Mean (± SD) *M. leidyi* abundances were 0.12 ± 0.21 and 0.19 ± 0.33 individuals m^−2^ with a maximum of 1 and 1.3 individuals m^−2^ for the Swedish and Danish surveys, respectively (Table 2 and 3). The mean oral-aboral size of *Mnemiopsis leidyi* was 21.8 ± 6 mm (range: 10 – 40 mm) for the Swedish and 20.1 ± 4.8 mm (range: 5.6 – 46 mm) for the Danish surveys, respectively (Table 2 and 3).

***Bolinopsis infundibulum*** (Ctenophora) were caught at 25 stations throughout the investigation area, but the species was more prevalent in the western and central part of the North Sea and the Kattegat. No animals have been caught in coastal Danish waters of the North Sea (Fig. 4). This ctenophore had an overall mean (± SD) and maximum abundance in the entire dataset of 0.04 ± 0.05 and 0.17 individuals m^−2^ or 0.96 ± 1 and 4 individuals 1000 m^−3^ (see raw data). Mean (± SD) *B. infundibulum* abundances were 0.02 ± 0.01 and 0.05 ± 0.05 individuals m^−2^ with a maximum of 0.04 and 0.17 individuals m^−2^ for the Swedish and Danish surveys, respectively (Table 2 and 3). Size ranged between 5 to 20 mm with a mean size of 14.8 ± 6 mm for the Swedish survey and between 19 to 63 mm with a mean size of 21.3 ± 2.2 mm for the Danish survey, respectively (Table 2 and 3).

***Aequorea vitrina*** (Hydrozoa) were caught at 23 stations in low densities (n = 30) throughout the sampling area apart from the Kattegat (Fig. 5A). This hydrozoan jellyfish had an overall mean (± SD) and maximum abundance in the entire dataset of 0.01 ± 0.005 and 0.02 individuals m^−2^ or 0.17 ± 0.09 and 0.47 individuals 1000 m^−3^ (see raw data). Mean abundance (± SD) and maximum was 0.01 ± 0.004 (max: 0.018) and 0.01 ± 0.01 (max: 0.023) individuals m^−2^ or 0.12 ± 0.05 (max: 0.23) and 0.25 ± 0.1 (max: 0.47) individuals 1000 m^−3^ for the Swedish and Danish surveys, respectively (Table 2 and 3). Average sizes (± SD) were 154 ± 31.5 mm (range: 100 – 220 mm) and 239 ± 58.5 mm (range: 110 - 300 mm) for the Swedish and Danish survey, respectively (Table 2 and 3).

***Tima bairdii*** (Hydrozoa) were caught at 60 stations throughout the entire investigated area, but the species was more abundant in the central and western part of the North Sea (Fig. 5A). The overall mean (± SD) and maximum abundance was 0.09 ± 0.2 and 1.4 individuals m^−2^ or 1.86 ± 3.6 and 25.9 individuals 1000 m^−3^ (see raw data). Separated by survey, mean (± SD) abundances were 0.01 ± 0.01 and 0.1 ± 0.2 individuals m^−2^ with a maximum of 0.02 and 1.4 individuals m^−2^ for the Swedish and Danish surveys, respectively (Table 2 and 3). Average (± SD) *T. bairdii* abundance 1000 m^−3^ was 0.22 ± 0.1 (max: 0.42) and 2.27 ± 3.9 (max: 25.9) for the Swedish and Danish surveys, respectively (see Table 2 and 3). Sizes ranged between 40 to 60 mm with a mean size of 49.2 ± 4.9 mm for the Swedish survey and 5 to 84 mm with a mean size of 46.6 ± 10.3 mm for the Danish survey, respectively (Table 2 and 3).

***Clytia* spp.** (Hydrozoa) was only counted in the Danish survey, and present at 12 stations. They were most abundant in the eastern part of the North Sea along the western coastline of Denmark (Fig. 5B), with a mean (± SD) and maximum abundance of 0.07 ± 0.06 and 0.24 individuals m^−2^ or 2.6 ± 2.46 and 9.35 individuals 1000 m^−3^ (Table 3). Size ranged between 2 and 45 mm, with an average of 7.5 ± 4.6 mm (Table 3).

***Leuckartiara octona*** (Hydrozoa) was only counted in the Danish survey and primarily present in the eastern part of the North Sea, along the entire west coast of Denmark (Fig. 5B). *L. octona* were caught at 31 stations with an overall mean (± SD) and maximum abundance of 0.06 ± 0.06 and 0.24 individuals m^−2^ or 2.11 ± 2.18 and 9.3 individuals 1000 m^−3^ (Table 3). Size ranged between 2.3 to 16 mm with an average size of 6.6 ± 1.25 mm (Table 3).

***Muggiaea atlantica*** (Hydrozoa, Order: Siphonophorae) was only encountered in the Danish survey and found primarily in the northeastern part of the North Sea (Fig. 5B). *Muggiaea atlantica w*ere caught at five stations and height ranged between 4 to 15 mm (only polygastric stages present and nectosome height measured). Mean (± SD) and maximum abundance were 0.26 ± 0.25 and 0.57 individuals m^−2^ or 5 ± 4.4 and 9.42 individuals 1000 m^−3^ (Table 3).

***Aglantha digitale*** (Hydrozoa) were quantified during both surveys. *A. digitale* was among the smallest, but most abundant species during the investigation in January to February 2021 in the North Sea. This hydrozoan jellyfish was found at 111 stations (>75.5 % of all stations) and was especially abundant in the eastern-central part of the North Sea (Fig. 6). The overall mean (± SD) abundance combining both surveys was 18 ± 39.5 (max: 204.8) individuals m^−2^ or 338 ± 655 (max: 3059) individuals 1000 m^−3^. Separated by survey, average (± SD) abundance was 3.18 ± 6.82 (max: 35) and 27 ± 47.5 (max: 205) individuals m^−2^ or 50.45 ± 110.2 (max: 628) and 506.6 ± 774 (max: 3059) individuals 1000 m^−3^ (Table 2 and Table 3). Average size (± SD) was 7.45 ± 1.46 mm throughout the entire Danish investigation area (Table 3).

While the Danish dataset consists of counts and sizes, the Swedish dataset grouped abundances and estimated densities based on four abundance groups, see methods for details.

***Cyanea* spp.** (Scyphozoa) was present at 59 stations during both surveys (Fig. 7). However, small ephyra stages were not quantified at the first 11 stations (station numbers 101-114) in the Kattegat during the Swedish Survey (see raw data table). *Cyanea* spp. were most abundant in the eastern and southern part of the North Sea (Fig. 7). Throughout both surveys, the overall average abundance (± SD) was 0.19 ± 0.27 individuals m^−2^ or 6.12 ± 10.12 individuals 1000 m^−3^. Mean (± SD) and maximum abundance were 0.08 ± 0.17 (max: 0.78) and 0.25 ± 0.3 (max: 1.44) individuals m^−2^ or 1.7 ± 3.2 (max: 13.3) and 8.6 ± 11.7 (max: 62.8) individuals 1000 m^−3^ (Table 3). Size ranged between 3 to 70 mm with an average size of 20 ± 8.4 mm during the Danish survey (Table 3), while only an overall average size was estimated to allow for weight extrapolations during the Swedish survey (Table 2).

## 2 Experimental Design, Materials and Methods

Samples were collected during the Danish and Swedish contributions to the International Bottom Trawl Survey (IBTS) on board the Danish R/V DANA (DTU Aqua, Denmark) and the Swedish R/V Svea (SLU, Sweden), respectively. The Danish sampling took place in the western, central and eastern part of the North Sea during February 2021, while the Swedish sampling took place in the north-eastern part of the North Sea as well as in the Skagerrak and Kattegat during January – February 2021. The goal and procedure for the IBTS survey [1,2] is bottom trawling during day-time to provide abundances and spatial distribution for a range of commercially important fish species together with CTD casts to describe the physical environment. Additionally, plankton sampling is conducted during night-time (approx. 17:00-5:00) on the IBTS, with the primary goal to catch herring larvae to provide a recruitment index for the stock assessment of North Sea autumn spawning herring, as well as to assess the ichthyoplankton community in general. This procedure was extended to include gelatinous macrozooplankton diversity and abundance data as they represent an important competitor as well as potential predator of fish larvae. Samples were collected at a total of 147 stations.

The same methodology [1,2] was used on the Danish and Swedish surveys, where gelatinous macrozooplankton was assessed from MIK net casts. This net consists of a large metal ring with a 2 m diameter opening and a 13 m long, 1.6 mm meshed net bag, which ends in a cod end. The last meter of the net bag and the cod end has a smaller mesh size of 500 µm. The net was hauled with a double oblique profile from the surface to c. 5 meters above the bottom (maximum depth of 100 m) at a ship speed of 3 knots, through the water. For further details about the MIK net and haul procedures, it is referred to the ICES MIK manual [2]. After each haul, the net was carefully retrieved, the hindmost part of the net was washed and the cod end stored in a chiller or cold sea water until analyses in the ship based wet laboratory. Upon analyses, the entire cod end content was analysed for gelatinous macrozooplankton and fish larvae on a light table, stereomicroscope (DK dataset) or under a magnifying lamp (Swedish dataset). For the Danish part, all jellyfish were identified to species or genera level, counted and measured to the nearest 0.01 mm with an electronic caliper, connected to a laptop. The Swedish survey identified all larger gelatinous zooplankton organisms >0.5 cm (excluding smaller Hydrozoan species apart from *A. digitale, T. bairdii* and *A. vitrina*), using a conventional caliper.

Calibrated flow meters in the centre of the net opening were used to assess the water volume filtered during the tow. The amount of filtered water in m^3^ was calculated from the Delta flow meter counts, divided by the flowmeter's calibration factor multiplied with the net opening area (3.142 m^2^) [3]. Abundance per m^−3^ was estimated by dividing the total species count per net with the filtered water volume. To estimate area specific abundances (individuals m^−2^), volume specific counts (individuals m^−3^) were multiplied with the sampling depth (m). Volume specific abundance data are presented per 1000 m^−3^ (Table 2 and 3). Length-weight regressions from literature were used to convert length, height or diameter (mm) to wet weight (g) for each species (Table 1).

For *Aequorea vitrina* and *Clytia* spp., dry weight (DW) was converted to wet weight (WW) assuming that DW represents 4 % of WW [4]. For *Leuckartiara octona,* a regression for the similarly shaped species *Sarsia tubulosa* (both belonging to the Order Anthoathecata) was used [5], assuming a carbon weight of 0.5 % of WW [6]. For *Muggiaea atlantica*, height ranged between 3.5 to 14.5 mm (only polygastric stages caught and nectosome height used) and an average wet weight value of 0.0195 g individuals^−1^ was taken for all sightings [7]. Volume regression for *Mnemiopsis leidyi* [8] was also used for *Bolinopsis infundibulum,* due to their similar morphology. Displacement volume (mL) was estimated from oral-aboral lengths (L_oa_) [8] and was converted to wet weight (g) assuming a specific weight ratio of 1.0 g cm^−3^, following earlier assumptions for *A. aurita* [9,10].

While the Danish dataset consists of counts and sizes, the Swedish dataset grouped abundances and estimated densities for the very abundance hydrozoan species *A. digitale*. To do so, densities were approximated into four abundance groups of 1-10 individuals (1+), 11-100 individuals (2+), 101-1000 individuals (3+) and > 1000 (4+). For abundance estimates and biomass conversions, average abundance for each group was set to 5, 50, 500 and 5000 for group 1+ to 4+, respectively. Based on size data from the Danish survey in the same area (see Fig. 1) we estimated an average size of 7.8 mm for *A. digitale* and used this average size for weight estimates of the Swedish dataset (Fig. 8). For the Danish dataset, sub-sampling was conducted for abundance estimations of *A. digitale* at few stations during the cruise. To confirm sub-sampling factors, samples from ten stations with the highest *A. digitale* densities were re-analysed in the laboratory. Comparing sub-sampling factors estimated during the cruise with the ones from the laboratory led to an overall negligible difference in total abundance estimates of 1.8 ± 3.2 % between both methods. Additionally, it is noted that for Swedish dataset, ephyra of *Cyanea* spp. were not encountered in large quantities during the first 11 stations (<5 individuals per station) and have therefore not been included in the database for station numbers 101-114 (see raw data table).

The spatial distribution of gelatinous macrozooplankton (Figs. 2-8) was visualized using the program QGIS 3.22.2 (QGIS.org, 2022. QGIS Geographic Information System. QGIS Association). Sampled stations were plotted according to their coordinates and abundance and wet weight of the different gelatinous macrozooplankton species are presented on a volume specific basis (1000 m^−3^, see Fig. 2, Fig. 3, Fig. 4, Fig. 5, Fig. 6, Fig. 7). Wet weight contributions of all major gelatinous macrozooplankton groups have been calculated and are presented on an area specific basis (Fig. 8). All data are available via the electronic supplement (Appendix 1) or the online repository Zenodo [17].

## Ethics Statements

Not applicable.

## Credit author statement

**Louise G. Køhler:** Data generation, curation, analyses, visualization; writing - first draft; **José Martín:** Data visualization, analyses; writing - final manuscript draft; **Bastian Huwer:** Conceptualization, methodology, data generation, analyses; writing – editing and commenting final draft; **Malin Werner:** Data generation, analyses; writing – editing and commenting final draft; **Maria Ovegård, Karolina Wikström** and **Anders Wernbo:** Data generation, reading final draft; **Cornelia Jaspers:** Conceptualization, methodology, data visualization, re-analyses; writing: editing first draft and writing final manuscript.

## Funding

This work was supported by the Villum und Velux Foundations [grant number 25512] to CJ.

## Declaration of Competing Interest

The authors declare that they have no known competing financial interests or personal relationships that could have appeared to influence the work reported in this paper.

## Data Availability

Jaspers,Huwer,Werner_Q1-IBTS-2021 (Original data) (Zenodo).

## Appendix Supplementary materials

Image, application 1
